# CMT-308, a Nonantimicrobial Chemically-Modified Tetracycline, Exhibits Anti-Melanogenic Activity by Suppression of Melanosome Export

**DOI:** 10.3390/biomedicines8100411

**Published:** 2020-10-13

**Authors:** Shilpi Goenka, Sanford R. Simon

**Affiliations:** 1 Department of Biomedical Engineering, Stony Brook University, Stony Brook, NY 11794-5281, USA; sanford.simon@stonybrook.edu; 2 Department of Biochemistry and Cellular Biology, Stony Brook University, Stony Brook, NY 11794-5281, USA; 3 Department of Pathology, Stony Brook University, Stony Brook, NY 11794-5281, USA

**Keywords:** chemically-modified tetracycline, CMT-308, tyrosinase, B16F10 cells, melanosome export, dendricity, HEMn-DP cells, coculture

## Abstract

CMT-308 is a nonantimicrobial chemically-modified tetracycline (CMT), which we have previously shown exhibits antifungal activity and pleiotropic anti-inflammatory activities, including inhibition of the enzymatic activity of matrix metalloproteinases (MMPs). Based on its chemical structure, we hypothesized that CMT-308 could inhibit melanogenesis and might be a candidate for the treatment of skin hyperpigmentation disorders which occur due to unregulated melanin biosynthesis and/or transport. CMT-308 was first studied for any effects on activity of the enzyme tyrosinase in vitro using a purified preparation of mushroom tyrosinase; the mode of inhibition of the soluble fungal enzyme was evaluated by Lineweaver-Burk and Dixon plots as well as by non-linear least squares fitting. Next, the effects of CMT-308 were tested in mammalian cell cultures using B16F10 mouse melanoma cells and further validated in darkly-pigmented human melanocytes (HEMn-DP). Our results showed that micromolar concentrations of CMT-308 inhibited mushroom tyrosinase enzyme activity, using the first two substrates in the melanogenesis pathway (l-tyrosine and l-3,4-dihydroxyphenylalanine (l-DOPA)); CMT-308 inhibited mushroom tyrosinase primarily via a mixed mode of inhibition, with the major contribution from a competitive mode. In B16F10 cell cultures, CMT-308 (10 µM) significantly diminished total melanin levels with a selective reduction of extracellular melanin levels, under both basal and hormone-stimulated conditions without any cytotoxicity over a duration of 72 h. Studies of potential mechanisms of inhibition of melanogenesis in B16F10 cells showed that, in mammalian cells, CMT-308 did not inhibit intracellular tyrosinase activity or the activity of α-glucosidase, an enzyme that regulates maturation of tyrosinase. However, CMT-308 suppressed MITF protein expression in B16F10 cells and showed copper chelating activity and antioxidant activity in a cell-free system. The significantly lower extracellular melanin levels obtained at 10 µM indicate that CMT-308’s anti-melanogenic action may be attributed to a selective inhibition of melanosome export with the perinuclear aggregation of melanosomes, rather than a direct effect on the tyrosinase-catalyzed steps in melanin biosynthesis. These results were validated in HEMn-DP cells where CMT-308 suppressed dendricity in a fully reversible manner without affecting intracellular melanin synthesis. Furthermore, the capacity of CMT-308 to inhibit melanosome export was retained in cocultures of HEMn-DP and HaCaT. In summary, our results offer promise for therapeutic strategies to combat the effects of hyperpigmentation by use of CMT-308 at low micromolar concentrations.

## 1. Introduction

Melanin, a biopolymeric pigment, is produced by melanocytes within specialized vesicles called melanosomes [1], which are exported to keratinocytes where they form a supranuclear cap believed to protect against UV damage by conversion of UV photons into heat [2,3,4]. Ultraviolet (UV) irradiation triggers the secretion of α-melanocyte stimulating hormone (α-MSH) from epidermal keratinocytes [5]; this hormone binds to the melanocortin 1 receptor (MC1R) on melanocytes and activates microphthalmia transcription factor (MITF), which in turn increases the expression of tyrosinase and other proteins [5,6]. Despite having other protective benefits of free-radical scavenging activity and toxic metal-ion chelation in addition to UV photoprotection, the aberrant production of melanin pigment in melanocytes and the over secretion of melanosomes from melanocytes can cause accumulation of melanin in keratinocytes, which manifests as hyperpigmentary disorders such as post-inflammatory hyperpigmentation (PIH), melasma, and lentigo senilis (LS) [7], which cause significant psychosocial stress as it compromises the aesthetic appearance. Furthermore, enhanced melanogenesis might be a risk factor for skin cancer, melanoma [8]. Hence, inhibitors of melanogenesis are also appealing as adjuvants for sensitizing melanoma cells to anticancer therapeutics [9] and to improve radiotherapy outcome [10,11]. Tyrosinase (EC 1.14.18.1) is the central rate-limiting enzyme in the melanogenesis pathway, as it catalyzes the hydroxylation of l-Tyrosine to l-3,4-dihydroxyphenylalanine (l-DOPA) as well its subsequent oxidation to Dopachrome [12,13]. As tyrosinase is a metalloenzyme with two copper ions; chelators that can sequester copper have also shown promise as a target for pigmentation disorders [14,15,16]. The intracellular transport of tyrosinase is regulated by copper uptake and the N-glycosylation process [17]; the role of the enzyme α-glucosidase has been implicated in this glycosylation process in previous reports [18,19]. Consequently, certain antidiabetic drugs such as voglibose [20] and acarbose [21], which are known to inhibit α-glucosidase activity, also suppress melanogenesis. The use of commercial depigmenting agents, hydroquinone (HQ), kojic acid (KA) and arbutin, has been limited since they inhibit tyrosinase in a manner that cannot be fully reversed [22] and cause deleterious effects such as erythema, contact dermatitis, genotoxicity, and carcinogenicity [23,24]. As the process of melanogenesis involves a multi-step pathway, compounds which can target different steps in this pathway provide an attractive target for melanogenesis inhibition. After synthesis and maturation within melanocytes, the melanosomes are exported to the neighboring keratinocytes via multiple molecular pathways [25]. The process of translocation of melanosomes to keratinocytes is accomplished by dendrites, which can retract or extend [26], thereby reducing or enhancing the export of melanosomes and modulating skin pigmentation [27,28]. Currently, several reports have documented novel compounds that have shown efficacy in reducing melanogenesis by inhibiting melanosome export through the reduction in dendricity [29,30,31]. Additionally, we have previously reported on a synthetic steroidal compound, asoprisnil [32], and a fungal-derived natural antibiotic, Thermorubin (TR) [33], both of which suppressed dendricity as one of the modes of inhibition of melanosome export. MITF, one of the key transcription factors which controls tyrosinase and other melanogenesis-related enzymes [34,35], also regulates melanocyte dendricity [36].

Chemically-modified tetracyclines (CMTs also referred to as COLs) belong to a group of novel synthetic tetracycline derivatives, which have the dimethylamino group at C4 carbon eliminated; while this elimination abolishes antimicrobial activity, the anti-MMP activity is retained or even enhanced; these CMTs showed pleiotropic inhibitory properties and were primarily developed as host-modulating agents by Golub et al. [37,38,39]. We have reported on CMT-308, a nonantimicrobial 9-amino derivative of CMT-3, which inhibited the zinc metalloproteinase activity of *Bacillus anthracis* lethal factor [40], in part through its capacity to chelate metal ions such as Zn^2+^. In addition, we have also demonstrated that CMT-3 displayed inhibitory activity towards serine proteinases in a previous patent [41]. Other studies have documented that CMT-308 has also shown anticancer potential against a variety of cancers including melanoma, prostate [42], lung, and breast cancer [43]. In addition to the anticancer therapeutic properties of CMT-308, another study described the potential of CMT-308 for the treatment of systematic mast cell disorder and rheumatoid arthritis by targeting mast cell proliferation [44].

Previous reports have documented that tetracycline and doxycycline, a semi-synthetic tetracycline, induce cutaneous pigmentation [45,46], but the effects of CMTs on epidermal pigmentation have remained largely unexplored. In the current study, we have selected CMT-308, the 9-amino derivative of CMT-3, because of its exceptional profile of biological actions when compared to other CMTs. For example, CMT-308 was shown to inhibit MMPs with a greater potency than the original 10 CMTs, with an IC_50_ of 1.5 µM against MMP-8 and an IC_50_ of 4.2 µM against MMP-9 [47]. In addition, CMT-308 has shown minimal phototoxicity as compared to other CMTs when tested using the standard 3T3 NRU phototoxicity test in our laboratory (unpublished results). CMT-308 bears structural similarity to chemically-modified curcumins (CMCs) [48,49], as they both share a β-diketone moiety that confers the capacity to bind Zn^2+^ ions. In our previous study, we have reported that the polycyclic antibiotic TR inhibited melanosome export in vitro [33]. As CMT-308 shares structural similarities, including a polycyclic structure containing a β-diketone moiety, to TR, we were prompted to test if CMT-308 might also exhibit anti-melanogenic efficacy. Hence, we hypothesized that CMT-308 might inhibit activity of the enzyme tyrosinase, as well as subsequent steps in the pathway of melanogenesis, that could form the basis for its potential use for the treatment of hyperpigmentation.

## 2. Materials and Methods

### 2.1. Materials

Synthetic melanin (Cat#: M8631), mushroom tyrosinase (Cat#: T3824), α-MSH, Baker’s yeast α-glucosidase, p-nitrophenyl β-d-glucopyranoside (PNG), l-ascorbic acid, kojic acid (KA), l-3,4-dihydroxyphenylalanine (l-DOPA), L-tyrosine, pyrocatechol violet (PV), copper sulfate, and Fontana Masson (FM) staining kit (Cat #: HT200-1KT) were purchased from Sigma-Aldrich (St. Louis, MO, USA). CMT-308 was a gift from Galderma (Switzerland, Europe). Dimethyl sulfoxide (DMSO; Cat# 196055) was obtained from MP Biomedicals LLC (Solon, OH, USA). MTS cytotoxicity assay (CellTiter 96^®^ AQueous One Solution Reagent) was procured from Promega Corporation (Madison, WI, USA). 2,2-diphenyl-1-picrylhydrazyl (DPPH) and 2′,7′-dichlorodihydrofluorescein diacetate (H_2_DCFDA) reagents were procured from Molecular Probes Inc. (Eugene, OR, USA). Penicillin-streptomycin (10,000 U/mL) antibiotic cocktail, Dulbecco’s modified Eagles medium (Gibco™ DMEM), Dulbecco’s phosphate buffered saline (DPBS), TrypLE Express enzyme (1×), hank’s buffered salt saline (HBSS; Hyclone™), and sodium hydroxide solution (NaOH, 1N) were procured from Thermo Fisher Scientific (Waltham, MA, USA). Heat-inactivated fetal bovine serum (HI- FBS) was purchased from R&D Systems Inc. (Minneapolis, MN, USA). Medium 254 and human melanocyte growth supplement (HMGS) were procured from Cascade Biologics (OR, USA). Cell lysis buffer (Cat #: EA-0001) was purchased from Signosis Inc. (Santa Clara, CA, USA) and fixative (16% paraformaldehyde aqueous solution) was procured from Electron Microscopy Sciences (Hatfield, PA, USA).

### 2.2. Mushroom Tyrosinase Activity Using *l*-TYR and *l*-DOPA Substrates

The direct effects of CMT-308 on tyrosinase enzyme activity were tested using a purified mushroom tyrosinase enzyme using l-TYR (monophenolase) and l-DOPA (diphenolase) as substrates. For assaying monophenolase activity, CMT-308 (80 µL) at different concentrations was diluted in 50 mM sodium phosphate buffer (pH 6.5) and was added to a 96-well plate followed by the addition of 100 µL of 0.5 mM l-TYR substrate solution. The reaction was initiated by the addition of 20 µL of 125 µg/mL mushroom tyrosinase enzyme and the reaction’s progress was monitored by measuring the kinetics of absorbance at 475 nm (for 20 min every 30 s). The slopes of the kinetic readings were calculated to determine and compare tyrosinase activity from control and expressed as % of the untreated control.

For assaying diphenolase activity, 80 µL of CMT-308, prepared at different concentrations in 50 mM sodium phosphate (pH 6.5) buffer, was added to a 96-well plate followed by the addition of 100 µL of freshly prepared 0.75 mM l-DOPA substrate solution. Subsequently, 20 µL of 35 µg/mL mushroom tyrosinase was added and the production of dopachrome was monitored by measuring the kinetics of absorbance at 475 nm (for 30 min every 30 s). The slopes of the kinetic readings were calculated, and results were expressed as % of control similar to the aforementioned method.

### 2.3. Kinetic Analysis of Enzyme Inhibition

In order to study the mechanism of tyrosinase inhibition by CMT-308, a kinetic study of monophenolase and diphenolase activities at multiple concentrations of substrates were undertaken. For the monophenolase activity assay, the final concentrations of l-TYR substrate selected were 0.125, 0.25, 0.5 and 1 mM, with the final concentration of mushroom tyrosinase enzyme as 12.5 µg/mL. In the case of diphenolase activity assay, the final concentration of enzyme was 3.5 µg/mL and the final concentrations of l-DOPA substrate were 0.375, 0.75, 1.5 and 3 mM. The slopes from the linear range of the progress curves of absorbance at 475 nm vs. time were recorded as apparent velocities and the inverse values, 1/v, were plotted vs. the inverse substrate concentrations at different fixed inhibitor concentrations based on the Lineweaver and Burk (L-B) method [50]. Dixon plots of the inverse velocities, (1/v), as a function of the inhibitor concentrations at different substrate concentrations were also constructed to further study the apparent mode of inhibition by CMT-308.

### 2.4. Copper Chelation Assay

Copper-ion chelation activity was evaluated using a pyrocatechol violet (PV) chromogenic reagent based on the method reported in our earlier work [51]. Briefly, 100 µL of different concentrations of CMT-308 were prepared using 50 mM sodium acetate buffer (pH 6.0) and aliquoted in a 96-well plate. Next, 10 µL of 2 mM copper sulfate solution was added and incubated for 10 min followed by the addition of 10 µL of 2 mM PV solution and further incubated for 20 min. The absorbance was read at 632 nm using a microplate reader and copper chelating activity was reported as % normalized to control.

### 2.5. α-Glucosidase Activity Assay

In order to test if CMT-308 might directly affect the α-glucosidase activity, we assayed the α-glucosidase activity using α-glucosidase purified from Baker’s yeast based on the method described previously [33]. Briefly, 80 µL of CMT-308 was prepared using 0.05 M phosphate buffer (pH 6.5) and was aliquoted to a 96-well microplate followed by the addition of 100 µL of 1.2 mM p-nitrophenyl-α-d-glucopyranoside (PNG) substrate. A total of 20 µL of 0.45 units of enzyme solution was added; the rate of the formation of p-nitrophenol was monitored at 405 nm for 15 min using the kinetic mode at 37 °C in a microplate reader. The enzyme activity was computed as: (Rate of sample reaction/Rate of control reaction) × 100%.

### 2.6. DPPH Radical Scavenging Assay

DPPH (2,2-Diphenyl-1-picryl-hydrazyl) is a stable free radical which, after reaction with antioxidant compounds that can donate a hydrogen atom, leads to a color change from violet to yellow which can be easily measured and has been widely used to assess antioxidant activities of compounds previously [52]. Briefly, DPPH was freshly prepared in methanol and mixed with different concentrations of CMT-308 in triplicates in a 96-well plate. The control group consisted of DPPH only and ascorbic acid (AA) at 2.5 µg/mL was used as a positive control. The final DPPH concentration was 100 μM and the final volume was 200 µL in the plate (20 µL samples with 180 µL of DPPH). The plate was covered and incubated for 30 min. Absorbance was read at 517 nm and the % DPPH radical scavenged was calculated based on this formula: = [(Ao − Ac)/Ao] × 100, where Ao is the absorbance of the control and Ac is the absorbance of the compound. The concentration of CMT-308 which scavenged 50% of DPPH radicals (half-maximal inhibitory concentration; IC_50_) was calculated from non-linear regression analysis of a dose–response curve.

### 2.7. Cell Culture

B16F10 melanoma cells (CRL-6475™) were procured from the American Type Culture Collection (ATCC, Manassas, VA, USA) and were cultured using DMEM supplemented with 10% HI-FBS and 1% antibiotics (penicillin-streptomycin). Human epidermal melanocytes from neonatal darkly-pigmented donor (HEMn-DP; lot# 1781055) were obtained from Cascade Biologics (Portland, OR, USA) and were cultured in Medium 254 supplemented with 1% human melanocyte growth supplement and 1% antibiotics. HEMn-DP cells were used between passages 5 and 10 for all assays. Human keratinocytes (HaCaT) cells were obtained from AddexBio (San Diego, CA, USA) and were cultured in DMEM with 10% HI-FBS and 1% antibiotics. For all cell cultures, we did not use amphotericin B as a supplement in the medium.

### 2.8. Cytotoxicity Assay

B16F10 cells were seeded at 5 × 10^3^ cells/well in 0.2 mL medium in a 96-well plate for 24 h, after which the culture medium was aspirated, and CMT-308 was added at various concentrations and further incubated for 72 h. At the end of 72 h, the culture medium was aspirated and replaced by 100 μL of fresh medium containing 20 μL of MTS reagent and incubated for 40 min. Subsequently, the absorbance was read at 490 nm using a Versamax^®^ microplate reader and cell viability was calculated from the absorbance values relative to control groups and expressed in %.

HEMn-DP cells (2 × 10^4^ cells/well in 0.2 mL medium) were inoculated in a 96-well plate and cultured for 48 h. After this, the culture medium was replaced with fresh medium containing CMT-308 at various doses (5–25 µM) and incubated for 72 h. After the treatment, MTS assay was conducted similar to the method above except an incubation period of 3 h was used. Data were expressed as % normalized to control.

### 2.9. Melanin Content Estimation

B16F10 cells were seeded at 5 × 10^4^ cells/well in 1.5 mL medium in 12-well plates and incubated for 24 h followed by the replacement of medium with fresh medium containing CMT-308 in the presence or absence of 100 nM α-MSH [53,54] and cultures maintained for a period of 72 h. KA (500 μM) was used as a positive control. At the end of treatment, the extracellular melanin was estimated by measuring absorbance of the culture supernatants at 475 nm using a microplate reader. For intracellular melanin assay, cells remaining in the wells were detached using TrypLE Express (1×; Gibco) and cell pellets were washed in PBS. After aspiration, 250 μL of 1N NaOH was added and heated to 70 °C to solubilize melanin. The absorbance of the lysate was read at 475 nm; a standard curve was made using synthetic melanin prepared at different concentrations in 1N NaOH and was used to quantify amount of both the intracellular and extracellular melanin which were subsequently reported in µg/mg protein for each group.

For melanin content estimation in HEMn-DP cells, 1.1 × 10^5^ cells were cultured in 12-well plates in 1.5 mL medium/well, cultured for 72 h followed by replacement of fresh medium containing CMT-308, and cultures were maintained for another 72 h. At the end of treatment, the intracellular melanin was quantified similarly to the method reported above.

### 2.10. Cellular Tyrosinase Activity 

B16F10 cells (2 × 10^4^ cells/well in 1 mL medium) were cultured in 24-well tissue culture plates for 24 h, and CMT-308 was then added in the presence or absence of 100 nM αMSH, and further incubated for 72 h. At the end of treatments, cells were detached, washed in PBS, and lysed in lysis buffer. Following lysis, they were centrifuged and 50 µL of lysates were then aliquoted in a 96-well microplate with the addition of 100 µL of 3 mM l-DOPA substrate solution. The absorbance was then measured at 475 nm in the kinetic mode setting every 30 s for 40 min at 30 °C using a microplate reader. The % tyrosinase activity was calculated from the slope of the linear range of the velocities of inhibition.

For the assessment of intracellular tyrosinase activity in HEMn-DP cells, we seeded 1.1 × 10^5^ cells/well in a 12-well plate and cultured for 48 h, after which CMT-308 was added, and cultures were maintained for a further 72 h. At the end of the treatments, cells were pelleted, lysed, and the tyrosinase activity was similarly measured in lysates as outlined above.

### 2.11. Intracellular α-Glucosidase Activity in B16F10 Cells

In order to test if CMT-308 might inhibit the activity of the enzyme α-glucosidase which regulates the maturation of tyrosinase enzyme in cells, we conducted experiments to assay for cellular *α*-glucosidase activity. B16F10 cells (1.2 × 10^5^ cells/well in 3 mL complete medium) were seeded in 6-well plates, the next day CMT-308 was added in the presence or absence of α-MSH, and cells were maintained for 72 h. At the end of 72 h, the cells were detached, washed in PBS, lysed, and centrifuged; 50 µL of supernatants were aliquoted in a 96-well plate followed by the addition of 100 µL of 2 mM PNG substrate solution; the rate of the formation of the reaction product: p-nitrophenol was monitored at 405 nm in kinetic mode for 45 min (with reading every 40 s) at 37 °C in a microplate reader. The intracellular α-glucosidase activity was calculated as (rate of sample reaction/rate of control reaction) × 100%.

### 2.12. Intracellular ROS Measurement in B16F10 Cells

We assessed the levels of intracellular ROS generation using the probe 2′,7′-dichlorodihydrofluorescein diacetate (H_2_DCFDA), which is well-established for the estimation of cellular H_2_O_2_ [55]. This probe is cell-permeable and forms 2′,7′-dichlorodihydrofluorescein (H_2_DCF) after deacetylation by cellular esterases; H_2_DCF is subsequently oxidized in the presence of ROS to generate highly fluorescent 2′,7′-Dichlorofluorescein (DCF). Briefly, B16F10 cells (2 × 10^4^ cells/well in 1 mL complete medium) were cultured in 24-well plates for 24 h, followed by treatment with CMT-308 in the presence or absence of α-MSH and cultures maintained for another 72 h. Following treatment, the cells were washed with HBSS, and incubated with 50 µM probe diluted in DMEM medium (phenol-red free, sodium pyruvate free and serum-free) for 30 min at 37 °C. Subsequently, the cells were washed with HBSS and the plate was read using a fluorescence microplate reader (Gemini EM Spectramax, Molecular Devices) set at excitation/emission wavelengths of 485/535 nm using the well-scan mode (5–21 points/well). The relative fluorescence units (RFU) of CMT-308 was expressed as % of the control samples.

### 2.13. Estimation of MITF Protein Levels in B16F10 Cells

We assayed MITF protein levels in B16F10 cells to identify if the suppression of melanosome export by CMT-308 might be mediated, at least in part, by the downregulation of MITF. To this end, we employed a cell-based MITF ELISA kit (LifeSpan Biosciences, Seattle, USA). Briefly, B16F10 cells were seeded in a 96-well plate (4.5 × 10^3^ cells/well in 0.2 mL medium) and, after 48 h, the medium was replaced by fresh medium containing CMT-308 in the presence/absence of α-MSH and cultures were maintained for a duration of 72 h. After 72 h, the wells were washed, fixed, and further steps were conducted based on the manufacturer’s protocol. The relative levels of MITF levels were expressed as % of the untreated control.

### 2.14. Quantification of Dendricity in HEMn-DP Cells

The effects of CMT-308 on melanosome export in HEMn-DP cells were estimated by quantitation of dendricity indices of cells. Briefly, DP cells were seeded in 12-well plates (1 × 10^4^ cells/well) and after 48 h, CMT-308 was added, and the cultures were continued for another 72 h. At the end of treatments, the cells were imaged using a Nikon Labphot microscope equipped with a digital camera and computer-interfaced NIS Elements 5.0 imaging software package. The lengths of each dendrite in a cell was manually traced and added to calculate total dendrite length (TDL) similar to previous methods [56,57]. The number of dendrites in each cell were manually counted from images and expressed as % of the control group. In addition, the number of cells which had more than 2 dendrites were counted and reported as % of the total number of cells; this parameter is similar to that reported in previous studies [58].

### 2.15. Quantification of Recovery of Dendricity in HEMn-DP Cells

In order to assess whether the effects on dendricity by CMT-308 might be reversed upon removal of the compound from cultures, we conducted an exposure and recovery study. Briefly, 1 × 10^4^ DP cells/well were plated in a 6-well plate and treated with CMT-308 for 72 h. After 72 h (exposure), the wells were imaged. After this step, the cells were washed with HBSS and the culture medium was replaced with fresh medium without CMT-308, and the cultures were continued for an additional duration of 9 d (with two medium renewals in between). At the end of 9 d, the wells were imaged (recovery) and both sets of images (exposure and recovery) were analyzed for quantitation of dendritic parameters which were outlined earlier.

### 2.16. HEMn-DP Cocultures with HaCaT Cells

#### 2.16.1. Fontana-Masson (FM) Staining

HEMn-DP cells were cocultured using the method reported previously [59] with some modifications. Briefly, HEMn-DP cells were seeded in a 6-well plate for 24 h, after which HaCaT cells which had been precultured in serum-free keratinocyte growth medium (SF-KGM, Gibco) were added at twice the seeding density and cultured for another 24 h. After this step, CMT-308 was added to cocultures in SF-KGM medium and cultures were continued for 72 h, after which the wells were fixed in 4% paraformaldehyde. Subsequently, the cells were stained to visualize the melanosomes in coculture using FM staining method described in previous studies with some modifications [60,61]. FM staining was conducted according to manufacturer’s instructions. Briefly, cells were treated with ammoniacal silver solution, followed by incubations in 0.1% gold chloride solution and 5% sodium thiosulfate. After washing with distilled water, cells were counterstained with nuclear fast red and bright-field images were taken from random fields in the wells and were studied for visualization of melanosome accumulation.

#### 2.16.2. Quantification of Dendricity in Cocultures

Next, we evaluated if the inhibition of dendricity obtained in melanocyte monocultures by CMT-308 might be retained in melanocyte cocultures with keratinocytes. To this end, we cocultured the cells using the method described earlier. Briefly, HEMn-DP cells (1 × 10^4^ cells/ well) were seeded in a 6-well plate for 24 h followed by the addition of HaCaT cells in SF-KGM, and, after 24 h, CMT-308 was added for 72 h in SFM. At the end of treatments, the wells were imaged, and dendrite morphology were analyzed similarly to the method adopted in monocultures.

### 2.17. Statistical Analysis

One-way analysis of variance (ANOVA) with Dunnett’s or Tukey’s post-hoc test was run using GraphPad Prism software (version 8.4.2, San Diego, CA, USA) when comparing three or more groups, whereas a students unpaired *t*-test was used when comparing two groups. Differences were considered statistically significant at *p* < 0.05. All data are reported as Mean ± SD.

## 3. Results

### 3.1. Effects of CMT-308 on the Activity of Mushroom Tyrosinase Enzyme for l-TYR and l-DOPA Substrates 

**Figure 1:**
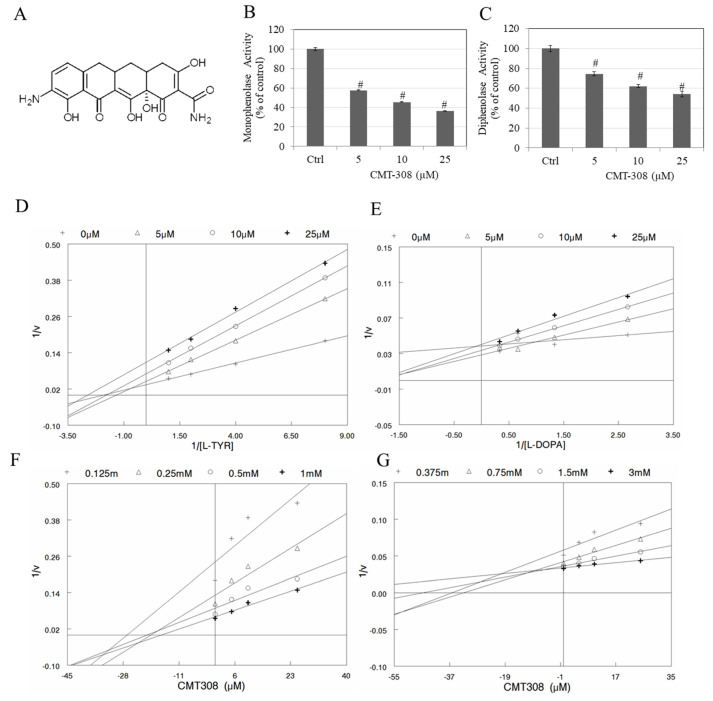
(**A**) chemical structure of CMT-308; (**B**) anti-monophenolase activity (using l-tyrosine (l-TYR) substrate); and (**C**) anti-diphenolase (using l-3,4-dihydroxyphenylalanine (l-DOPA) substrate) activity of CMT-308 using purified mushroom tyrosinase enzyme; # *p* < 0.01 vs. Ctrl, one-way ANOVA, Dunnett’s test; data are mean ± SD of triplicate determinations; mechanisms of inhibition were studied using Lineweaver-Burk plots for (**D**) l-TYR and (**E**) l-DOPA substrates as well as Dixon plots for (**F**) l-TYR substrate and (**G**) l-DOPA substrate.

We first tested if CMT-308 (chemical structure; Figure 1A) exhibits any inhibitory effect on the activity of mushroom tyrosinase enzyme using two substrates (l-TYR: monophenolase activity and l-DOPA: diphenolase activity). Our results showed that CMT-308 inhibited monophenolase activity of the soluble fungal tyrosinase with a robust inhibition of 42.46%, 54.47%, and 63.89% at 5, 10, and 25 µM, respectively (Figure 1B). The IC_50_ value of monophenolase activity inhibition was calculated to be 8.91 µM. Our results further showed that CMT-308 inhibited diphenolase activity of the soluble fungal enzyme by 25.37%, 37.93%, and 45.68% at 5, 10, and 25 µM, respectively (Figure 1C). The IC_50_ value of diphenolase activity inhibition was calculated to be 20.92 µM. Collectively, these results reveal that CMT-308 was 2.34-fold more potent in suppressing monophenolase activity of mushroom tyrosinase as compared to diphenolase activity.

### 3.2. Kinetic Analysis of Mushroom Tyrosinase Inhibition by CMT-308

We next tested the mechanism of mushroom tyrosinase inhibition by both substrates (l-tyrosine and l-DOPA) using linear classical linearized Lineweaver-Burk (L-B) and Dixon plots. The nature of the intersection of lines can provide clues to the type of inhibition. For the inhibition of monophenolase activity by CMT-308, the results of the analysis of the L-B plot (Figure 1D) and Dixon plot (Figure 1F) revealed that CMT-308 does not inhibit activity by a purely competitive mode since all the lines did not intersect on the Y-axis. Furthermore, the mode was not that of a pure uncompetitive inhibitor since the lines were not parallel to each other. Additionally, the mode was not that of pure noncompetitive inhibition, as all lines did not intersect on the x-axis. Taken together, the mode of inhibition of monophenolase activity appears to be mixed.

In the case of analysis of the mechanism of inhibition of diphenolase activity by CMT-308, our results from L-B plots (Figure 1E) and Dixon plots (Figure 1G) showed that CMT-308 appears to be a mixed inhibitor of this reaction as well, as the patterns of inhibition were not those of pure competitive, pure noncompetitive inhibition, or pure uncompetitive inhibition.

Inhibitory parameters were also computed using a nonlinear least-squares fit of the kinetic data to three models of classical inhibition—competitive, noncompetitive, and uncompetitive—for each of the two substrates. Our results showed that while mushroom tyrosinase showed somewhat greater affinity for l-DOPA than l-tyrosine, CMT-308 was an inhibitor of both the monophenolase and diphenolase activities of the enzyme (Table S1). The mode of inhibition for both activities appears to be mixed, with the major contribution from a classic competitive mode. This predominance of a classic competitive mode was especially evident for inhibition of diphenolase activity, which was inhibited by CMT-308 binding in a competitive mode with an apparent affinity for the enzyme (Ki) approximately one order of magnitude greater than that for binding in a noncompetitive or uncompetitive mode (Table S1). Collectively, these data indicate that CMT-308 is a mixed inhibitor for both substrates, with a predominant component of competitive inhibition, due to which it may be capable of binding both to the free enzyme and, with significantly lower affinity, to the enzyme-substrate complex (ES complex).

### 3.3. Effects of CMT-308 on Copper Chelating Capacity 

**Figure 2:**
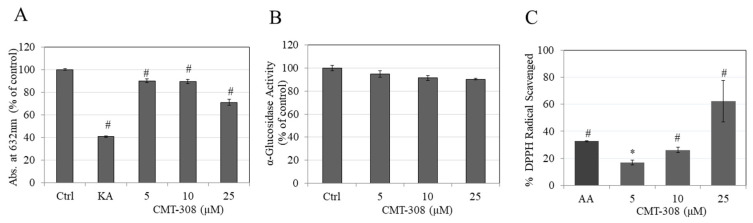
(**A**) Copper chelation assay of CMT-308 measured by reduction in absorbance at 632 nm; kojic acid (KA) at 0.5 mM was used as the positive control; (**B**) effect of CMT-308 on α-glucosidase enzyme activity, measured with pNG substrate; (**C**) antioxidant activity of CMT-308 measured by DPPH radical scavenging assay with ascorbic acid (AA) used as positive control at 2.5 µg/mL (~14 µM); * *p* < 0.05 and # *p* < 0.01 vs. Ctrl; one-way ANOVA with Dunnett’s test; data are mean ± SD of triplicate determinations.

As tyrosinase contains copper in its active site, we next tested if CMT-308 may chelate copper, which was tested using the chromogenic substrate, PV. Our results showed that CMT-308 showed a significant copper chelating activity of 10%, 11%, and 29% at concentrations of 5, 10, and 20 µM, respectively (Figure 2A).

### 3.4. Effects of CMT-308 on α-Glucosidase Activity

Next, we tested if CMT-308 may have any direct inhibitory effect on the activity of the enzyme α-glucosidase in a cell-free assay, as this activity is important in the delivery of tyrosinase to melanosome membranes; however, there was no alterations in levels of enzyme activity on treatment with CMT-308 (Figure 2B).

### 3.5. Effects of CMT-308 on DPPH Radical Scavenging Activity

Our results showed that CMT-308 is a strong scavenger of DPPH radical in a dose-dependent manner; CMT-308 at concentration 5, 10, and 25 µM scavenged DPPH radical by 15.24%, 23.86%, and 56.77%, respectively (Figure 2C). The mean value of IC_50_ of DPPH radical inhibition by CMT-308 was calculated to be 21 µM. These results demonstrate that the antioxidant capacity of CMT-308 was comparable to the positive control ascorbic acid which scavenged 29.81% at the concentration of 2.5 µg/mL (~14 µM).

### 3.6. Effects of CMT-308 on Total Melanin in B16F10 Cell Cultures under Both Basal and Hormone-Stimulated Conditions 

**Figure 3:**
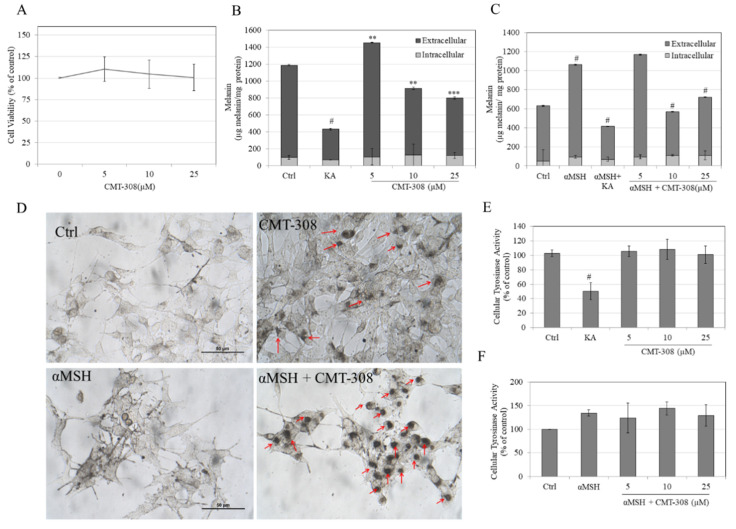
(**A**) Viability of B16F10 cells exposed to different concentrations of CMT-308 over 72 h assessed by MTS assay; total melanin levels in the cultures were quantified and were expressed as extracellular and intracellular melanin levels under conditions of (**B**) basal melanogenesis and (**C**) melanocyte-stimulating hormone (α-MSH)-stimulated melanogenesis; KA was used as a positive control at a concentration of 500 µM; (** *p* < 0.01; *** *p* < 0.001; # *p* < 0.0001 vs. Ctrl; one-way ANOVA with Dunnett’s test); (**D**) representative photomicrographs of B16F10 cells showing control and CMT-308 (10 µM) treated cells for basal and α-MSH-stimulated melanogenesis; red arrows show the perinuclear aggregation of melanosome granules which was noticeable in CMT-308 treated groups; intracellular tyrosinase activity in B16F10 cellular lysates under (**E**) basal and (**F**) α-MSH-stimulated conditions (# *p* < 0.01 vs. Ctrl; one-way ANOVA with Tukey’s test); all data are mean ± SD of at least three independent experiments except for (**B**,**C**), which is representative of one independent experiment performed on triplicate samples.

We next conducted cellular assays using B16F10 mouse melanoma cell model to study if CMT-308 could demonstrate anti-melanogenic activity in cell cultures. To this end, CMT-308 was first tested for cytotoxicity; our results showed that CMT-308 was nontoxic to B16F10 cells over the concentration range 5–25 µM for the duration of 72 h (Figure 3A), hence these concentrations were used for further experiments.

Under the conditions of basal melanogenesis (i.e., no added α-MSH), treatment with CMT-308 at 5 µM significantly increased total melanogenesis (i.e., the sum of intracellular and extracellular melanin levels) by 22% but at concentrations of 10 µM and 20 µM suppressed total melanogenesis by 23% and 33%, respectively (Figure 3B). Treatment with KA (500 µM) suppressed total melanogenesis by 64% (Figure 3B). In the case of α-MSH-stimulated melanogenesis, treatment with α-MSH alone significantly increased melanogenesis by 68% compared to untreated control (baseline—100%), while cotreatment with α-MSH and CMT-308 at 5 µM resulted in a slight increase of only 17% over the unstimulated control (non-significant); however, cotreatment with α-MSH and higher concentrations of CMT-308 significantly suppressed total melanogenesis by 78% at 10 µM and by 54% at 20 µM, as compared to α-MSH group (Figure 3C). Cotreatment of α-MSH group with KA (500 µM) suppressed total melanin levels by 103%, as compared to α-MSH group.

The morphology of B16F10 cells after α-MSH stimulation showed a visibly higher dendritic appearance (Figure 3D) than that seen in cells maintained under basal conditions, while co-treatment with α-MSH and CMT-308 at 10 µM resulted in perinuclear aggregation of melanosomes; some perinuclear aggregation of melanosomes in the presence of CMT-308 alone was also observed in cells maintained under basal conditions. Collectively, these results indicate that CMT-308 exhibits enhanced antimelanogenic activity in the B16F10 cells stimulated with α-MSH, with potent suppression of total melanogenesis in the presence of CMT-308 concentrations of 10 µM or greater.

### 3.7. Effects of CMT-308 on α-Glucosidase Activity in B16F10 Cells 

**Figure 4:**
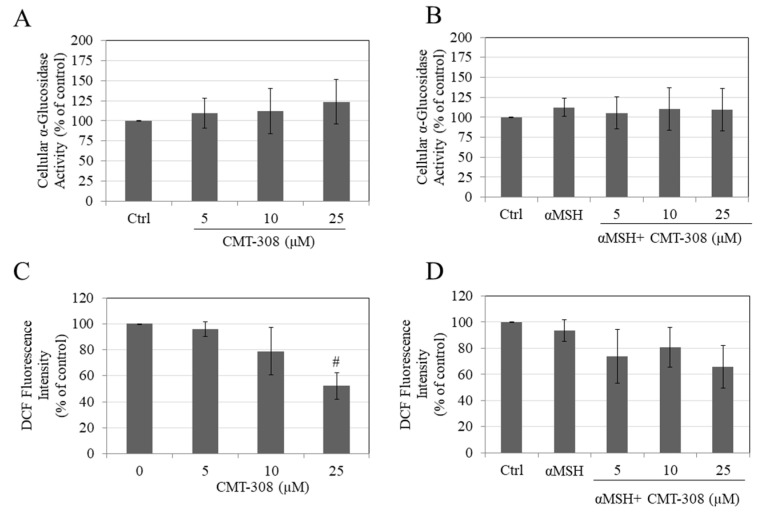
Intracellular α-glucosidase activity in B16F10 cells under (**A**) basal and (**B**) α-MSH-stimulated conditions; intracellular ROS generation measured by DCF fluorescence intensity under (**C**) basal and (**D**) αMSH stimulated conditions, # *p* < 0.01 vs. Ctrl; all data are mean ± SD of at least two independent experiments.

Since α-glucosidase plays a role in the maturation of tyrosinase in mammalian cells, we next tested if CMT-308 might inhibit activity of this enzyme in cellular lysates. Our results showed that CMT-308 did not affect the α-glucosidase activity at any concentration, either under basal (Figure 4A) or under α-MSH-stimulated conditions (Figure 4B), indicating that other mechanisms may be involved in its anti-melanogenic action.

### 3.8. Effects of CMT-308 on Intracellular ROS Generation in B16F10 Cells 

Our results showed that CMT-308 at a concentration of 25 µM significantly attenuated ROS generation by B16F10 cells maintained under basal conditions (Figure 4C). In cells maintained in the presence of α-MSH, CMT-308 showed a trend for reduction in ROS generation, although no significance was reached at any concentration (Figure 4D).

### 3.9. Effects of CMT-308 on MITF Protein Expression in B16F10 Cells under Hormone-Stimulated Conditions 

**Figure 5:**
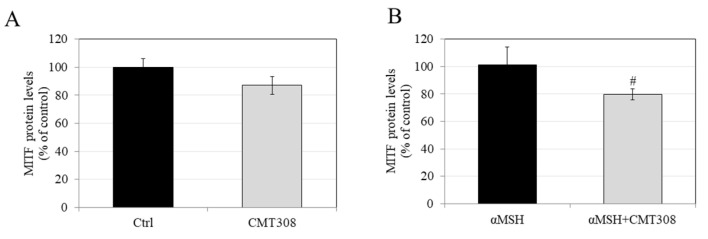
Effects of CMT-308 on MITF protein expression in B16F10 cells under (**A**) basal and (**B**) α-MSH-stimulated conditions; data are mean ± SD of three replicates; # *p* < 0.01 vs. αMSH; unpaired-student’s *t*-test.

Our results showed that, under basal conditions, CMT-308 at 10 µM showed a trend for reduction in MITF protein levels (by 12.92%), which did not reach significance (Figure 5A), while it significantly attenuated MITF levels by 21.56% (Figure 5B) under α-MSH-stimulated conditions. Altogether, these results are consistent with the idea that the inhibition of melanogenesis by CMT-308 under α-MSH-stimulated conditions may be related, at least in part, to downregulation of MITF expression.

### 3.10. Effects of CMT-308 on Melanin Synthesis and Cellular Tyrosinase Activity in HEMn-DP Cells 

**Figure 6:**
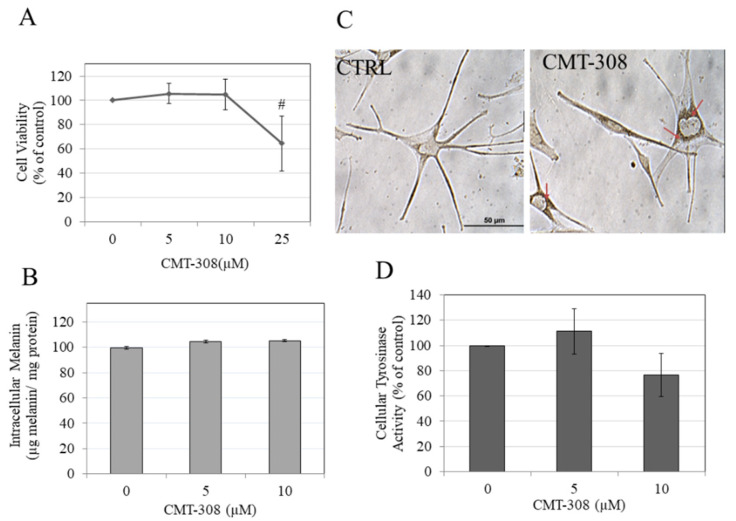
(**A**) human epidermal melanocytes-darkly pigmented (HEMn-DP) cell viability after treatment with CMT-308 for 72 h as measured by MTS assay; # *p* < 0.01 vs. Ctrl, One-way ANOVA with Dunnett’s test; data are mean ± SD of three independent experiments; (**B**) intracellular melanin levels in HEMn-DP cells treated with CMT-308 for 72 h; data are mean ± SD of a representative independent experiment on triplicate samples; (**C**) representative bright-field images of HEMn-DP cells in control (Ctrl) and CMT-308 (10 µM)-treated group; red arrows show perinuclear aggregation of melanosomes in the CMT-308-treated group; objective magnification 40×; (**D**) tyrosinase activity in HEMn-DP cellular lysates after treatment with CMT-308 for 72 h; data are mean ± SD of at least two independent experiments.

We next evaluated if CMT-308 might display anti-melanogenic activity in human melanocytes obtained from darkly-pigmented skin. To this end, CMT-308 was first assessed for cytotoxicity over a duration of 72 h in HEMn-DP cells. Our results showed that CMT-308 induced significant toxicity at 25 µM (mean cell viability was diminished by 40%, *p* < 0.01), while concentrations of 5 and 10 µM were nontoxic (Figure 6A); these concentrations were used for further analysis.

CMT-308 did not affect intracellular melanin levels in HEMn-DP cells at 5 or 10 µM (Figure 6B). Interestingly, we noted a distinctive aggregation of melanosomes in the perinuclear region in melanocytes treated with CMT-308 at 10 µM; in contrast, melanosomes in the control group could be seen in both the cytoplasm and dendrites (Figure 6C). This observation confirmed that CMT-308 appears to inhibit melanosome transport and is similar to the findings obtained earlier in B16F10 mouse cells.

Next, our results on the effects of CMT-308 on tyrosinase activity of HEMn-DP cellular lysates showed a trend for reduction of activity by 23.53% (Figure 6D) at 10 µM, but this inhibition did not reach statistical significance.

### 3.11. Effects of CMT-308 on Melanocyte Dendricity

**Figure 7:**
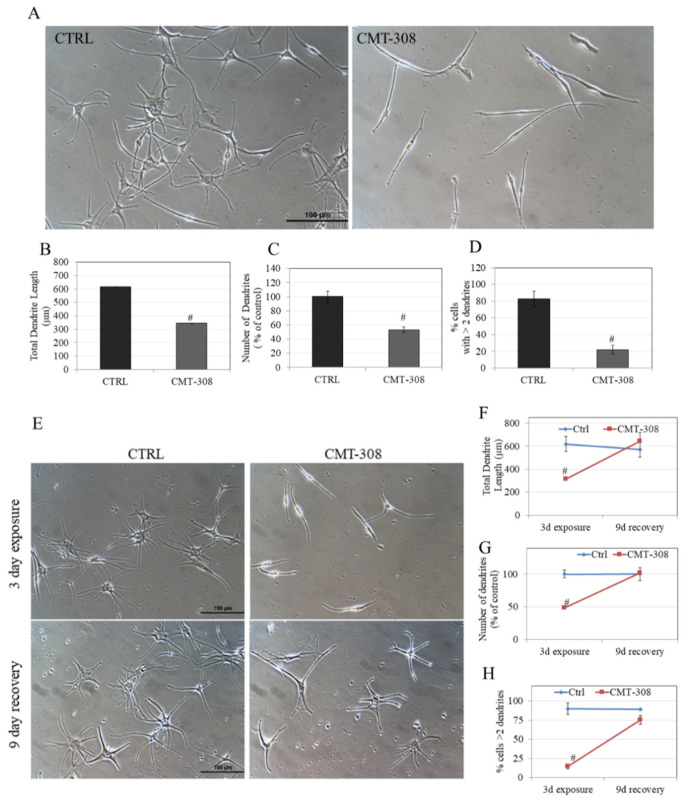
(**A**) Representative phase-contrast micrographs of HEMn-DP cells of Ctrl (0.4% DMSO) and CMT-308 (10 µM)-treated groups; quantification of melanocyte dendricity by (**B**) total dendrite length; (**C**) number of dendrites; (**D**) % cells with >2 dendrites; # *p* < 0.01 vs. Ctrl; (**E**) representative phase-contrast micrographs of HEMn-DP cells showing exposure and recovery of Ctrl and CMT-308-treated groups; quantification of dendricity by (**F**) total dendrite length; (**G**) number of dendrites; and (**H**) % cells with >2 dendrites; # *p* < 0.01 vs. Ctrl; data is mean ± SD of one representative experiment in triplicates with a total of 50–60 cells counted per group.

Melanosomes are exported from melanocyte tips to keratinocytes via dendrites from melanocyte tips; a reduction in dendrite number and/or length hinders this transport leading to hypopigmentation. The typical morphology of HEMn-DP cells is arborized with several dendrites; this was visibly reduced to mostly bipolar dendrites upon exposure to 10 µM CMT-308 (Figure 7A). Next, quantification of various indices of dendricity revealed that CMT-308 significantly reduced TDL by 43.80% (Figure 7B) as well as the number of dendrites by 46.74% (Figure 7C). Additionally, CMT-308 altered the distribution of cells with different dendrites; the % of cells with >2 dendrites were significantly diminished by 60.60% (Figure 7D). Collectively, this data showed that CMT-308 is a potent suppressor of melanosome export at the low micromolar concentration of 10 µM, as reflected in its diminution of all the parameters of melanocyte dendricity.

### 3.12. Reversibility Study of Melanocyte Dendricity by CMT-308 

The effects of a 72 h exposure to CMT-308 (10 µM) on melanocyte morphology, characterized by a drastic reduction in multi-dendritic morphology, were visibly reversed after replacement of the culture with CMT-308-free medium: After an additional 9 d of culture in CMT-308-free medium, the dendrites appeared similar in number and size to the recovery control group (Figure 7E).

We also quantified the dendricity parameters and our results demonstrate that, whereas exposure of HEMn-DP cells to CMT-308 (10 µM) showed an expected significant reduction in TDL, after the recovery period, the TDL reverted to baseline levels (recovery control; Figure 7F). A similar recovery was noted for the number of dendrites, which was reduced in the CMT-308-treated groups after 3 d but fully recovered to baseline control values after 9 d in CMT-free medium (Figure 7G). Lastly, the parameter of % cells with >2 dendrites, which was significantly reduced in CMT-308-treated cells after a 72 h exposure, also completely recovered to control after culture for 9 d in CMT-308-free medium (Figure 7H). Collectively, these results showed that the inhibitory effects of CMT-308 on melanosome export were fully reversible upon continued culture after removal of the compound from the culture medium.

### 3.13. Effects of CMT-308 on Dendricity in Melanocyte Cocultures 

**Figure 8:**
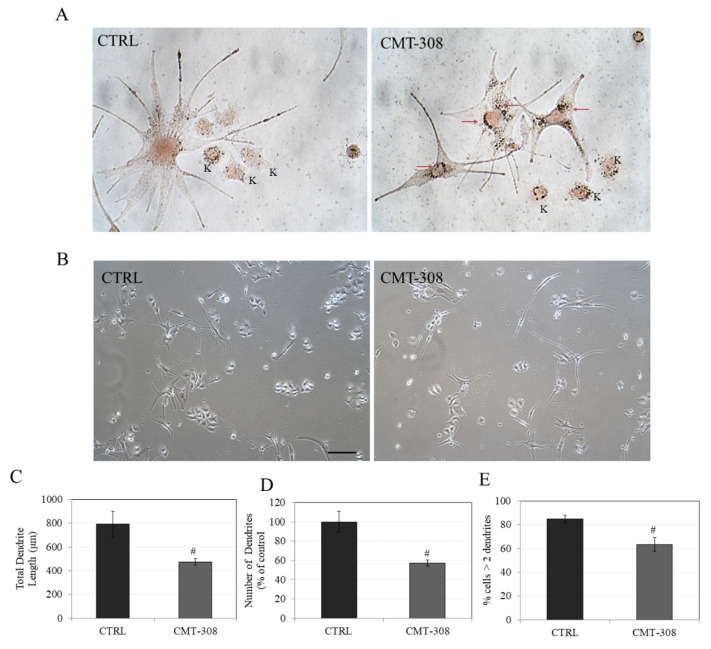
(**A**) Representative Fontana-Masson stained images of HEMn-DP-HaCaT coculture at 40× magnification under bright-field illumination; red arrows show dark-colored melanosomes aggregated around nucleus in CMT-308 (10 µM)-treated group; K: Keratinocytes; (**B**) representative phase-contrast micrographs of cocultures in Ctrl and CMT-308 (10 µM)-treated group; scale bar corresponds to 100 µm; quantitation of dendricity in cocultures of HEMn-DP: (**C**) Total dendrite length; (**D**) Number of dendrites; (**E**) % cells with >2 dendrites; # *p* < 0.01 vs. Ctrl, students unpaired *t*-test; data are mean ± SD from one representative experiment in triplicates; a total of 50–60 cells were counted per group.

As a single melanocyte is in contact with several keratinocytes in the epidermis, we tested if the inhibition of dendricity by CMT-308 observed in melanocyte monoculture might be retained in cocultures, which would support our conclusion that the inhibitory effect of CMT-308 on melanosome transfer appears to affect melanocytes alone and is not diminished when melanocytes are cultured in the presence of keratinocytes. CMT-308 was screened for cytotoxicity before proceeding with coculture experiments and was found to be nontoxic to keratinocytes over the tested concentration range (Figure S1). Our results showed that, in cocultures, CMT-308 (10 µM) exhibited a similar effect on melanocyte dendricity as that obtained in monocultures, with the appearance of perinuclear clustering (Figure 8A) and bipolar dendrites (Figure 8B). Next, the dendricity indices in the cocultures were quantified using the same parameters as those used in analyzing the monocultures; our results showed that CMT-308 significantly inhibited TDL by 40% (Figure 8C) and reduced the number of dendrites by 42.87% (Figure 8D). Furthermore, the % of cells with >2 dendrites were also significantly reduced by 21.6% (Figure 8E). Taken together, our results confirm that the capacity of CMT-308 to inhibit the machinery of melanosome export by reducing dendricity is retained in melanocyte cocultures.

## 4. Discussion

Our results demonstrate the novel finding that CMT-308 can suppress melanogenesis by targeting one or more steps of melanosome export in the melanogenesis pathway after the tyrosinase-catalyzed reactions. In addition to its capacity to suppress melanosome export in mammalian cell-based assays, CMT-308 also demonstrates potent inhibition of the first two reactions in the pathway of melanin synthesis catalyzed by a soluble preparation of fungal tyrosinase, functioning as a mixed inhibitor with a predominant competitive mode. CMT-308 also can chelate copper and inhibit ROS production in a cell-free system. Moreover, CMT-308 downregulated MITF protein levels and induced perinuclear aggregation of melanosomes, both of which may contribute to its capacity to attenuate melanin secretion. Interestingly, CMT-308 did not have any effect on the levels of melanin within cultured mouse or human melanocyte cell lines, which indicates that the mode of action of CMT-308 uniquely reflects a selective inhibition of melanosome export in the absence of effects on melanin synthesis. To the best of our knowledge, this is the first report of a synthetic CMT derivative which may function to reduce skin pigmentation via a novel mode by inhibiting melanosome export without affecting melanin biosynthesis.

Tetracyclines have been shown to exhibit a much higher affinity for Cu^2+^ than Zn^2+^ [62]. Our results on the copper-chelating activity of CMT-308 is in agreement with the cation-chelating capacity of CMTs [63,64]. By chelation of Cu^2+^ ions, CMT-308 might block the function of the essential metal in tyrosinase and could; thus, interfere with the process of tyrosinase-catalyzed melanin formation, if it could be shown that the tetracycline could have access to the enzyme in vivo. Furthermore, our results showed that, in a cell-free assay, CMT-308 exhibited antioxidant activity that might be mediated by the interaction of ROS with the phenolic hydroxyls of CMT-308; this is similar to the mechanisms of antioxidant activity of the antibiotic minocycline [65]. Steric hindrance due to the polyphenolic nature of the ring system of CMT-308 and intramolecular hydrogen bonding (IHB) involving the 9-amino group might contribute to ROS scavenging by stabilizing the phenolic radical. We used B16F10 mouse melanoma cells because this cell model has been well-validated as a robust model to screen for compounds which can diminish extracellular as well as intracellular levels of melanin. Our results in B16F10 cells demonstrated that CMT-308 was more effective in reducing total melanin levels under α-MSH stimulation than basal conditions, indicative of a stimulus-dependent enhanced inhibition of at least one step in melanogenesis. Reports have documented that culturing B16F10 cells in DMEM is not a true basal condition for expression of melanogenic activity, as l-tyrosine in the medium may mimic the effects of pro-melanogenic stimulus (α-MSH) inducing melanogenesis [66,67,68]. However, this induction is insignificant when compared to that achieved by α-MSH itself. Despite the capacity to exhibit antioxidant activity in an in vitro cell-free system, the reduction in ROS levels generated by B16F10 cells maintained in the presence of α-MSH and CMT-308 did not reach statistical significance, although a significant reduction in ROS levels generated in cells under basal conditions in the presence of 25 µM CMT-308 was detected. This discrepancy might be ascribable to differences in the actual profiles of detected analytes in both assays: The cellular ROS assay measured DCF while the cell-free assay measured DPPH radicals. Consequently, the direct comparison of cell-free and cellular effect need to be interpreted with caution. Differences in lipophilicity, cellular permeability, and cellular uptake might also explain the lack of effects in cell culture, as has been shown for CMTs previously [69]. Our results of the absence of effects on intracellular tyrosinase activity in both B16F10 and HEMn-DP cells stand in marked contrast to the results of potent suppression of mushroom tyrosinase activity obtained in the cell-free assay. This apparent discrepancy might be ascribed to the source of the tyrosinase purified from mushroom which exhibits distinct molecular differences from human tyrosinase [22] and different substrate specificities and catalytic activities [70]. However, mushroom tyrosinase remains as a popular reagent due to its ready availability and low cost. The caveats of relying solely upon data obtained with the fungal enzyme must be acknowledged. Similarly, our assays on the effects of CMT-308 on α-glucosidase were conducted using an enzyme preparation derived from yeast, which has been shown to have some structural differences from the mammalian enzyme [71]. Finally, we have used synthetic melanin as a standard for quantitation of intracellular melanin in lysates and extracellular melanin in culture media, but it exhibits some differences from natural melanin generated by cells, hence might introduce a systematic bias in our measurements [72].

Unlike B16F10 cells, which secrete copious amounts of melanin in the culture medium in a short timeframe, facilitating the study of extracellular melanogenesis-modulating agents, human melanocytes do not secrete comparable quantities of melanin into the medium. Consequently, we have employed measurements of dendricity as a surrogate marker for melanosome export in human melanocytes [33]. Our results of inhibition of melanosome export by CMT-308 bear some similarity to those obtained with TR, except CMT-308 appears to be more effective at inhibiting melanosome export without accumulation of intracellular melanin, unlike TR. Our results of reduction in dendricity are similar to those reported for the compound centauridein [31]. Our observations of perinuclear aggregation of melanosomes by CMT-308 are similar to the findings in previous reports where compounds, 16-kauren-2-beta-18, 19-triol [73], 2-methyl-naphtho[1,2,3-de] quinolin-8-one [74], and wogonin [75] suppressed the export of melanosomes by inducing their perinuclear aggregation. Melanosome aggregation and melanocyte dendritogenesis have been described as arising from the opposing actions of kinesin and dynein on melanosome trafficking, but they can be uncoupled by certain interventions [76]. There have been reports where dendritogenesis was suppressed without any alterations in melanosome distribution [31,33], while, in other cases, melanosome distribution was altered (perinuclear aggregation) in the absence of any changes in dendritic morphology, as seen in melanocytes of mice carrying the “dilute” mutation [77] and chick with “lavender” mutation [78]. Under both conditions though, melanosome export was disrupted leading to hypopigmentation. Our results of CMT-308 in this study indicate the involvement of both phenomena *viz.* alteration in melanosome distribution with perinuclear clustering and reduction of dendricity. In our recovery experiments, we noticed a complete reversal of suppressed dendricity and no substantial perinuclear aggregation. However, further experiments to evaluate the effects of CMT-308 on disruption and distribution of melanosomes in the presence of inhibitors of actin filaments, microtubules, or molecular motor proteins involved in melanosome trafficking will be necessary to dissect whether the inhibition of export is linked to cytoskeletal rearrangement or is independent of it.

A previous study compared the compound thiamidol with HQ and showed that thiamidol fully reversed inhibited melanin production, while HQ’s effects were irreversible in HEMn-DP cell cultures [22], although no information on melanosome export or dendricity was reported. Our results in HEMn-DP cell cultures showed that the diminution in dendricity by CMT-308 was fully reversible after its removal from the culture medium; this is an important prerequisite for its safety as a potential agent for the treatment of hyperpigmentary disorders, especially since disruption of melanosome export due to perinuclear clustering has been a hallmark of the Griscelli skin disorder [79]. Other studies have also documented the reversibility of inhibited melanosome export with compounds, wogonin [75], platycodin D [80], and niacinamide [81]. In particular, our results of recovered dendricity are similar to the compound platycodin D, in which the authors demonstrated recovery of dendricity based on qualitative evaluation [80]. Rac and Rho pathways regulate the export of melanosomes along dendrite tips [82]. Whether the inhibition of melanosome export by CMT-308 might be related to the involvement of these pathways was not assessed; however, future studies to evaluate them are warranted.

The inhibition of melanosome export by CMT-308 was further validated in a coculture model with melanocytes and keratinocytes in direct contact enabling cross-talk and transfer of pigment under conditions that may be expected to replicate those in vivo. Our results of a significant reduction of dendrite number, total dendrite length, and proportion of dendritic cells are similar to that of another study conducted with cocultures of melanocytes from burn patients where a similar attenuation of dendricity was noted [83]. We have also quantified melanosome transfer in cocultures after FM-staining based on previous methods [59,83]; however, we found no reduction in the number of melanosomes in keratinocytes of CMT-308-treated groups as compared to control after 72 h (Figure S2). We speculate that this might be due to the absence of an external stimulus in the coculture conditions employed in the current study, because of which melanosome transfer might not have been sufficiently stimulated, although we obtained an increase in TDL in cocultures as compared to monocultures. This reasoning is further supported by our results with B16F10 cell cultures, where CMT-308 showed a higher efficacy to inhibit melanosome export under α-MSH stimulation. Pigmentation in cocultures in the absence of an external stimulus is prolonged and can take >10 d [84]; external factors such as UVB irradiation or α-MSH are necessary to stimulate melanogenesis and melanosome transfer in HEMn-DP cocultures [85], although HEMn-DP cells are unresponsive to α-MSH-stimulation in monocultures [86]. Moreover, a recent study demonstrated that a mild stimulus involving a combination of l-tyrosine and NH_4_Cl stimulated pigmentation in HEMn-DP cocultures within a short duration of 5 d [87]. Further studies employing the use of such coculture methods with external stimulators to validate whether CMT-308 could in fact suppress the transfer of melanosome in recipient keratinocytes are warranted.

Several reports have documented that keratinocytes not only modulate melanocyte proliferation and dendricity [88,89], but also possess differential capacities to phagocytose and distribute melanosomes [90,91,92]; keratinocytes from light skin have melanosomes clustered perinuclearly, while those from dark skin have single melanosomes dispersed throughout cytoplasm [93,94]. Although we found no change in number of melanosomes transferred to keratinocytes in CMT-308-treated group, as compared to control, the hypothesis that CMT-308 might alter the arrangement of melanosomes in keratinocytes cannot be ruled out and would be interesting to explore in future studies by the ultrastructural analysis of transferred melanosomes in keratinocytes. We have used the spontaneously immortalized HaCaT cell line instead of primary keratinocytes to establish cocultures, since they have been previously used for cocultures [59] and provide a convenient model as they retain all the functional differentiation properties and markers of primary keratinocytes [95] without the challenges of limited proliferative capacity and changes in differentiation markers with the increasing passage [96,97]. Additionally, the difficulty to obtain skin phototype-matched keratinocytes poses another limitation. Our coculture experiments were conducted using a ratio of 1:2, which is different from the physiological ratio of 1:36 in vivo [98]. Thus, whether the efficacy of CMT-308 to inhibit melanogenesis is retained in a more physiological model such as a skin-tissue equivalent needs to be evaluated in future studies.

Rok et al. have reported that tetracycline [99], doxycycline [100], oxytetracycline [101] and chlortetracycline [102] show toxicity and alter the antioxidant status of HEMn-DP cells, but only after UVA exposure. They used a broad range of concentrations (2.5–250 µM) of these tetracycline derivatives in HEMn-DP cells (without UVA treatment) and reported lack of effects on melanin synthesis and tyrosinase activity, while melanosome export/dendricity at nontoxic concentrations was not reported. Although, their results are similar to our results of CMT-308 obtained at concentrations <25 µM, CMT-308 also suppressed dendricity indicative of its action as an inhibitor of hyperpigmentation, which is in contrast to the aforementioned tetracyclines, that induce hyperpigmentation, which is likely related to their capacity to stimulate melanin synthesis under UVA irradiation, as a part of a phototoxic reaction. Doxycycline has been previously shown to bind to melanin, which may explain its phototoxicity, although its binding affinity was weaker after UV exposure [103]. Although we have not evaluated the phototoxicity of CMT-308 in UVA irradiated HEMn-DP cells as it is beyond the scope of work, we speculate that CMT-308 might be less phototoxic to melanocytes than other tetracyclines; however, further rigorous studies to address this hypothesis are warranted. Previous studies in our laboratory have shown that CMT-308 exhibits lesser phototoxicity as compared to doxycycline when tested using 3T3 neutral red uptake test system [47]. We have not evaluated the melanin-binding capacity of CMT-308, which should be also investigated in future studies, especially if CMT-308 is administered in vivo. The advantages of CMTs include a balance of hydrophilicity and hydrophobicity, with generally acceptable stability and solubility profile, in addition to the absence of antimicrobial activity with fewer side-effects than antimicrobial tetracyclines. CMTs have shown to inhibit invasion and metastasis of human melanoma cells in vitro and in vivo in a previous report [104]. The significance of the discovery of CMT-308 as a skin depigmenting agent with a unique mechanism of inhibition of melanosome export lies not only in the identification of the apparent novel anti-melanogenic activity of this class of compounds, but also in the potential use of CMTs to be used as adjuvants for depigmenting melanomas in concert with other depigmenting agents. However, since CMT-308 has not been yet tested in humans, further studies are warranted before the clinical potential of this compound can be met.

## 5. Conclusions

In summary, our results demonstrate the novel anti-melanogenic activity of the chemically-modified tetracycline analog, CMT-308, which was validated in B16F10 mouse melanoma cells and HEMn-DP monocultures and cocultures. CMT-308 inhibited both steps of the reaction catalyzed by the soluble enzyme, mushroom tyrosinase. In addition, our research findings uncovered that CMT-308 exhibits a novel mechanism of inhibition of melanogenesis by exclusively targeting melanosome export with the induction of perinuclear aggregation without affecting intracellular melanin biosynthesis. Future studies to test the molecular mechanisms of downregulation of melanosome export by CMT-308 and whether the capacity to inhibit melanogenesis is retained in skin-tissue equivalents are warranted. Furthermore, studies to test the potency of other CMT analogs for their capacity to inhibit pigmentation are currently underway. Moreover, future studies to dissect the structure-activity relationship of CMTs with antimicrobial tetracycline and their derivatives (doxycycline, oxytetracycline, chlortetracycline) would be also interesting.

## Supplementary Materials

Supplementary materials can be found at https://www.mdpi.com/2227-9059/8/10/411/s1. Table S1: Kinetic parameters of inhibition of mushroom tyrosinase by CMT-308; Figure S1: MTS cytotoxicity assay for evaluating cellular viability of HaCaT cells treated with CMT-308 for 72 h; data are mean ± SD of two independent experiments; ** *p* < 0.01 vs. Ctrl, one-way ANOVA with Dunnett’s test. Figure S2: (**A**) Representative FM-stained images of HEMn-DP cocultures of untreated (Ctrl) and CMT-308 (10 µM)-treated group for 72 h; 40× objective magnification; (**B**) quantitation of melanosome particles in keratinocytes; data are mean ± SD of triplicates; no significance was found by student’s *t*-test.

## Abbreviations

PIH	Post-Inflammatory Hyperpigmentation
LS	Lentigo Senilis
KA	Kojic Acid
HQ	Hydroquinone
l-DOPA	l-3,4-dihydroxyphenylalanine
CMT	Chemically-Modified Tetracycline
MMP	Matrix Metalloproteinase
DMEM	Dulbecco’s Modified Eagle Medium
HI-FBS	Heat-Inactivated Fetal Bovine Serum
DMSO	Dimethyl Sulfoxide
MSH	Melanocyte Stimulating Hormone
HMGS	Human Melanocyte Growth Supplement
HEMn-DP	Human-Epidermal Melanocytes-Darkly Pigmented
TDL	Total Dendrite Length
DCF	Dichlorofluorescein
ANOVA	Analysis of Variance
MITF	Microphthalmia Transcription Factor
HBSS	Hank’s Balanced Salt Solution
ROS	Reactive Oxygen Species
ELISA	Enzyme Linked Immunosorbent Assay
FM	Fontana-Masson
Ctr	Control
IC_50_	Half-Maximal Inhibitory Concentration
