# Supplementary material for: CMT-308, a Nonantimicrobial Chemically-Modified Tetracycline, Exhibits Anti-Melanogenic Activity by Suppression of Melanosome Export
Source: Biomedicines. 2020 Oct 13;8(10):411. doi: 10.3390/biomedicines8100411 (PMC7601524; doi:10.3390/biomedicines8100411)
Supplement: Supplementary file 1 [file biomedicines-08-00411-s001.pdf]

SUPPLEMENTARY INFORMATION

CMT-308, a nonantimicrobial chemically-modified tetracycline, exhibits anti-melanogenic activity by suppression of melanosome export

Shilpi Goenka[1, #] and Sanford R. Simon[1,2,3]

[1] Department of Biomedical Engineering, Stony Brook, NY, 11794-5281-NY, USA.
[2] Department of Biochemistry and Cellular Biology, Stony Brook, NY, 11794-5281-NY, USA.
[3] Department of Pathology, Stony Brook University, Stony Brook, NY, 11794-5281-NY, USA.
* Correspondence: shilpi.goenka@stonybrook.edu

I. Mechanism of inhibition using non-linear curve fitting.

Non-linear curve fitting using a modified Levenberg-Marquardt algorithm was used to further analyze the apparent potency of CMT-308 for both substrates and the data were fitted to models of pure competitive, uncompetitive, and noncompetitive inhibition using the Enzfitter software (version 2.0, Biosoft, Cambridge, UK). For the inhibition of monophenolase activity, curve-fitting to a model of pure competitive inhibition gave an apparent value of Ki of 4.11 μM for CMT-308, while a fit to pure noncompetitive inhibition gave an apparent Ki value of 11.12 μM and a fit to pure uncompetitive inhibition gave an apparent Ki value of 5.34 μM (Table S1).

For the inhibition of diphenolase activity, curve-fitting to a model of pure competitive inhibition gave an apparent Ki value of 6.47 μM, while a fit to a model of pure uncompetitive inhibition gave an apparent Ki value of 34.15 μM and a fit to a model of pure noncompetitive inhibition gave an apparent Ki value of 43.12 μM (Table S1).

Table S1: Kinetic parameters of inhibition of mushroom tyrosinase by CMT-308 with two substrates

	**Monophenolase (L-TYR)**			**Diphenolase (L-DOPA)**		
**Inhibition Mechanism**	**Ki (μM)**	**Km (mM)**	**Vmax (mM/min)**	**Ki (μM)**	**Km (mM)**	**Vmax (mM/min)**
Competitive	4.11	0.37	24.34	6.47	0.22	31.48
Noncompetitive	11.12	0.52	28.01	43.12	0.40	35.57
Uncompetitive	5.34	0.75	32.95	34.15	0.46	36.32

K_i- Inhibition constant; K_m- Michaelis constant; V_{max}- maximum velocity

II. Cytotoxicity to human keratinocytes.

For testing cytotoxicity of CMT-308 with keratinocytes (HaCaT cells), 6 x10^3 cells/well were plated in a 96-well plate for 24 hours after which CMT-308 was added and cultures were continued for 72 hours. After this, culture medium was replaced by 20 µL of MTS dye with 100 µL of culture medium and the plate was incubated at 37°C for 1 hour. Subsequently, 100 µL volumes were aliquoted into a new 96-well plate and the absorbance was read at 490 nm using a microplate reader. The cellular viability was reported as % normalized to untreated controls.

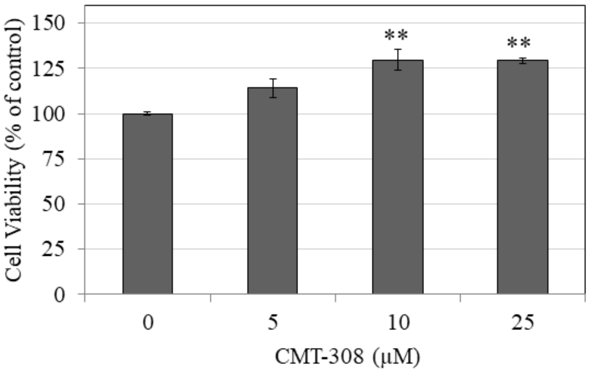

Figure S1: MTS cytotoxicity assay for evaluating cellular viability of HaCaT cells treated with CMT-308 for 72 h; Data are mean ± SD of two independent experiments; ***p* < 0.01 vs. Ctrl, One-way ANOVA with Dunnett's test.

III. Melanosome transfer assay in Fontana Masson (FM)-stained cocultures of HEMn-DP cells.

HEMn-DP cells were cocultured with HaCaT cells in contact coculture and stained using FM stain based on the method already described in the main text (under section 2.16.1). The number of melanosome particles transferred to keratinocytes in proximity to melanocytes were counted based on the dark-colored melanosomes which could be easily visualized under bright-field microscopy. Only the keratinocytes which were close to melanocytes and showed the presence of dark-colored melanosome particles were selected and the number of particles per keratinocyte were manually counted (with up to a total of 60 keratinocytes were scored in each treatment group) and the results were presented as % of untreated control.

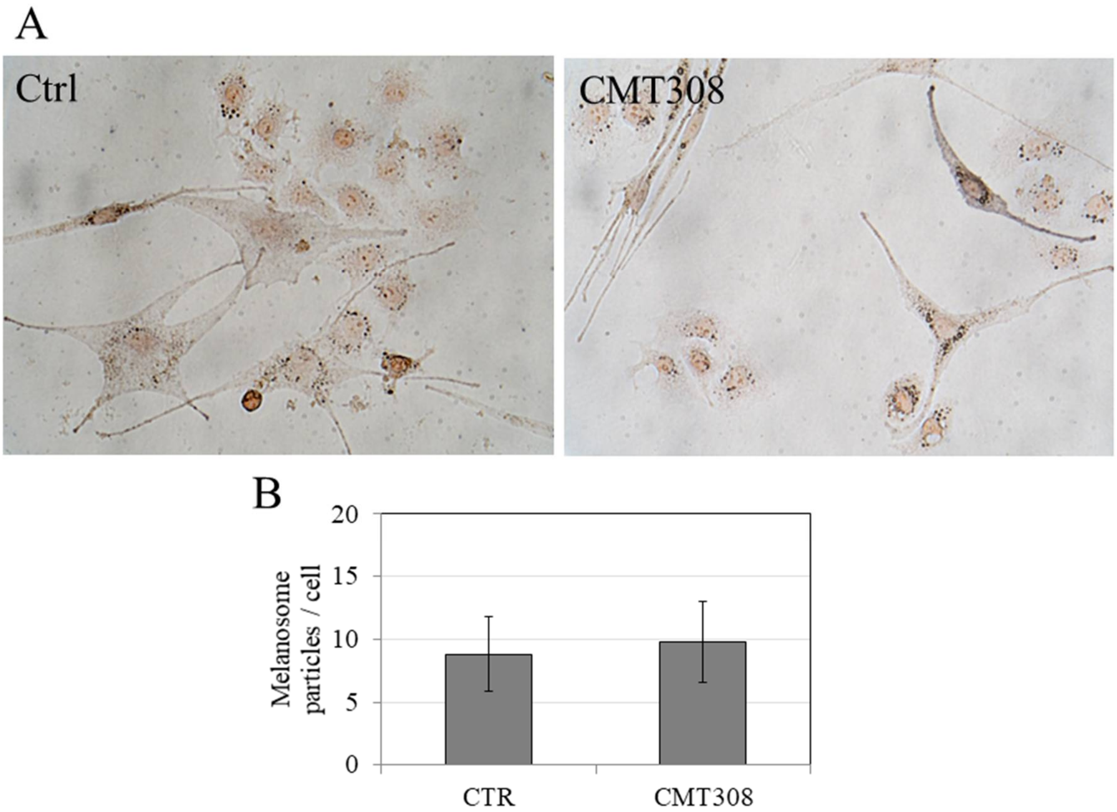

Figure S2: (**A**) Representative FM stained images of HEMn-DP cocultures of untreated (Ctrl) andCMT-308 (10 μM) -treated group for 72 h; 40× objective magnification; (**B**) Quantitation of melanosome particles in keratinocytes; Data are mean ± SD of triplicates; no significance was found by students t-test.
